# Cognitive Health in Massachusetts, New Hampshire, and Rhode Island: Findings From the Healthy Aging Data Reports

**DOI:** 10.1093/geroni/igaa057.307

**Published:** 2020-12-16

**Authors:** Richard Chunga, Taylor Jansen, Chae Man Lee, Shuangshuang Wang, Haowei Wang, Nina Silverstein, Frank Porell, Beth Dugan

**Affiliations:** 1 University of Massachusetts Boston, Boston, Massachusetts, United States; 2 University of Massachusetts Boston, Boston, United States; 3 Shandong University, Jinan, Shandong, China; 4 University of Massachusetts Boston, Needham, Massachusetts, United States

## Abstract

Over time persons with Alzheimer’s disease (AD) have impaired health, lower quality of life, and increased mortality compared to those without AD. This study describes state and community rates of Alzheimer’s disease, self-rated cognitive difficulty, and the % of the population age 85+ in three New England states (MA, NH, RI). Data sources were the American Community Survey (2009-2013 RI, 2012-2016 MA/NH) and the CMS Medicare Current Beneficiary Summary File (2012-2013 RI, 2015 MA/NH). Small area estimation techniques were used to calculate age-sex adjusted community rates for Alzheimer’s disease and related dementias (ADRD), self-reported cognitive difficulties, percentage of older adults 85 years or older, and the percentage of adults age 65+ living alone. State rates (range) were: AD: RI 14.4% (8-23%), MA 13.6% (6-19.31%), and NH 12% (5.49-33.51%). Self-reported cognitive difficulty: MA 8.3% (0-25.16%), RI 7.8% (2-18%), and NH were 6.9% (0-34.21%). Adults 85 years and older: RI 17.6% (6-24%), MA 15.2% (0-32.23%), and NH 12.9% (0-27.91%). Living alone: RI 30.4% (12-45%), MA 30.2% (6.25-50%), and NH 26.1% (6.13-72.55%). While there was significant variation across states, Rhode Island had the highest state rate of ADRD, older adults 85 and older, and percentage of older adults living alone. Within-state disparities among AD rates, cognitive difficulties, and living alone was highest in NH, but MA had the largest variation for community rates of adults 85+. Understanding the prevalence of brain health is important to policy and practice efforts to promote age-friendly communities. This research was supported by the Tufts Health Plan Foundation.

